# Supplementary figures and images for: The ITS region provides a reliable DNA barcode for identifying reishi/lingzhi (Ganoderma) from herbal supplements
Source: PLoS One. 2020 Nov 12;15(11):e0236774. doi: 10.1371/journal.pone.0236774 (PMC7660467; doi:10.1371/journal.pone.0236774)

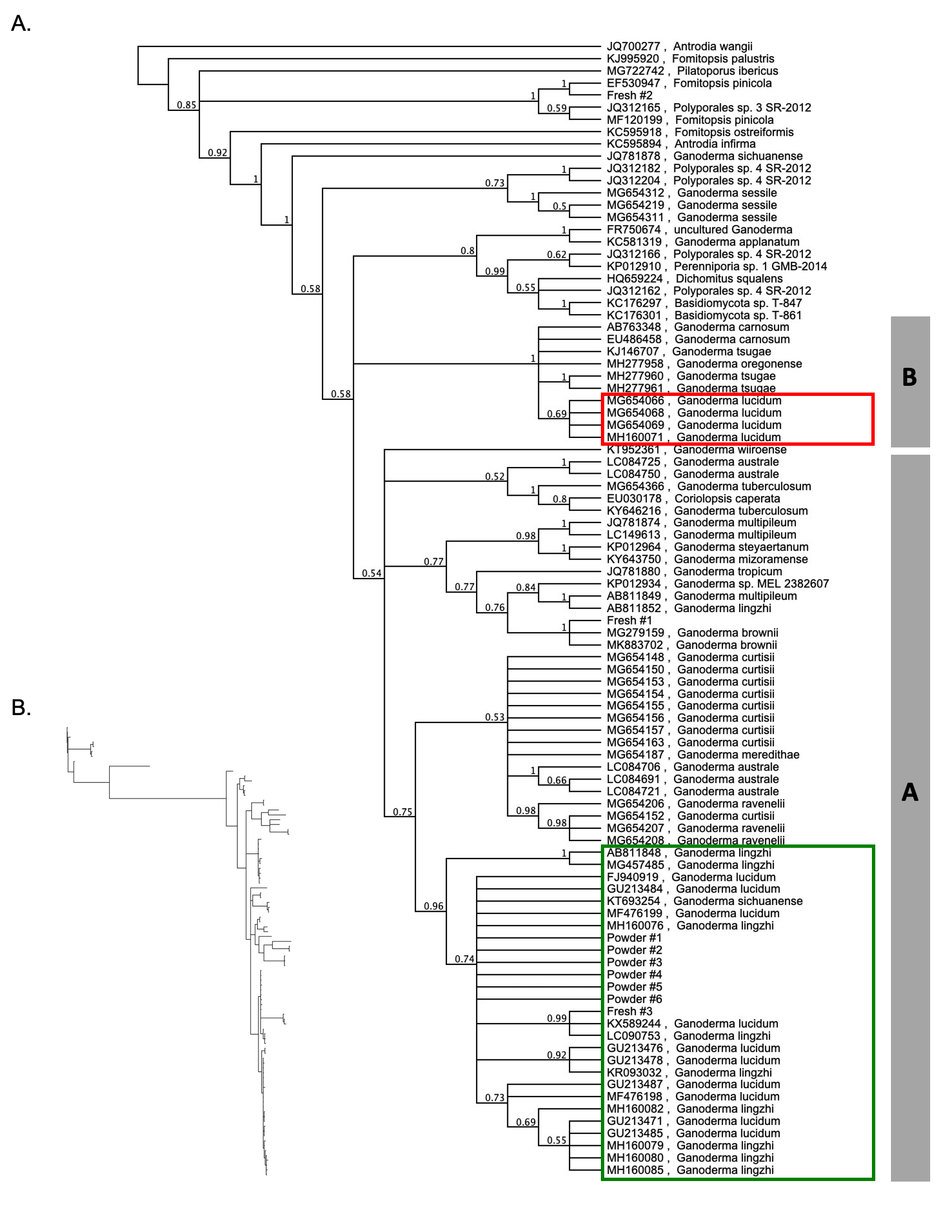

Supplement: S1 Fig — (A) Bayesian cladogram with Genbank accession numbers preceding species names for the reference panel. Samples are identified with reference to Table 1. Posterior probabilities greater than 0.50 are indicated along the branches. Branches with less than 0.50 posterior probability have been collapsed. Clade names A and B are from Loyd et al. [22] and Zhou et al. [24]. The red rectangle identifies the true G. lucidum samples per Loyd et al. [14] and the green rectangle contains the samples referred to as the G. lingzhi clade (many G. lucidum sequences are misidentified G. lingzhi). (B) Bayesian phylogram with unlabeled tips in the same order depicting branchlengths proportional to substitutions per site. (TIFF) [file pone.0236774.s001.tiff]
