# Supplementary material for: The ITS region provides a reliable DNA barcode for identifying reishi/lingzhi (Ganoderma) from herbal supplements
Source: PLoS One. 2020 Nov 12;15(11):e0236774. doi: 10.1371/journal.pone.0236774 (PMC7660467; doi:10.1371/journal.pone.0236774)
Supplement: S1 Table — (DOCX) [file pone.0236774.s002.docx]

**S1 Table. Three ITS primer pairs tested for amplification from reishi herbal supplements.**

| **Primer name** | **Direction^1^** | **Sequence** | **Reference** |
| --- | --- | --- | --- |
| ITS5 | F | GGA AGT AAA AGT CGT AAC AAG G | White et al. 1990 [37] |
| ITS4 | R | TCC TCC GCT TAT TGA TAT GC | White et al. 1990 [37] |
| G-ITS-F1 | F | ACC CTG TCG CTG AGA ACT TGA | Cao et al. 2012 [23] |
| G-ITS-R2 | R | TTG AGA GCG CAT CAC AAA GC | Cao et al. 2012 [23] |
| ITS1F | F | CTT GGT CAT TTA GAG GAA GTA A | Gardes & Bruns 1993 [50] |
| ITS4B | R | CAG GAG ACT TGT ACA CGG TCC AG | Gardes & Bruns 1993 [50] |

**^1^** F = forward; R = reverse
