# Supplementary material for: The ITS region provides a reliable DNA barcode for identifying reishi/lingzhi (Ganoderma) from herbal supplements
Source: PLoS One. 2020 Nov 12;15(11):e0236774. doi: 10.1371/journal.pone.0236774 (PMC7660467; doi:10.1371/journal.pone.0236774)
Supplement: S2 Table — (DOCX) [file pone.0236774.s003.docx]

**S2 Table. Genbank reference panel sampling.**

| **Genbank Accession** | **Taxon^1^** | **Location^2^** | **Reference** |
| --- | --- | --- | --- |
| KC595894 | *Antrodia infirma* | Finland | Ortiz-Santana et al., 2013 |
| JQ700277 | *Antrodia wangii* | China | Spirin et al., 2013 |
| KC176297 | *Basidiomycota sp. T-847* | USA: Michigan | Thorn et al., 1996 |
| AB811849 | *Basidiomycota sp. T-861* | USA: Michigan | Thorn et al., 1996 |
| KC176301 | *Basidiomycota sp. T-861* | USA: Michigan | Thorn, Unpublished |
| EU030178 | *Coriolopsis caperata* | Panama | Bergemann et al., Direct Submission |
| HQ659224 | *Dichomitus squalens* | Russia | Miettinen and Rajchenberg, 2012 |
| KC595918 | *Fomitopsis ostreiformis* | Indonesia | Ortiz-Santana et al., 2013 |
| KJ995920 | *Fomitopsis palustris* |  | Kirker and Blodgett, Unpublished |
| MF120199 | *Fomitopsis pinicola* | Antarctica | Vasilenko et al., Unpublished |
| EF530947 | *Fomitopsis pinicola* | Canada | Denis et al., Unpubished |
| KC581319 | *Ganoderma applanatum* | Canada | Berbee and Bazzicalupo, unpublished |
| LC084721 | *Ganoderma australe* | Malaysia:Sarawak, Lambir Hills National Park | Yamashita and Hirose, 2016 |
| LC084725 | *Ganoderma australe* | Malaysia:Sarawak, Lambir Hills National Park | Yamashita and Hirose, 2016 |
| LC084691 | *Ganoderma australe* | Malaysia:Sarawak, Lambir Hills National Park | Yamashita and Hirose, 2016 |
| LC084706 | *Ganoderma australe* | Malaysia:Sarawak, Lambir Hills National Park | Yamashita and Hirose, 2016 |
| LC084750 | *Ganoderma australe* | Malaysia:Sarawak, Lambir Hills National Park | Yamashita and Hirose, 2016 |
| MK883702 | *Ganoderma brownii* | USA: California, Anthony Chabot Regional Park | A. Rockefeller unpublished |
| MG279159 | *Ganoderma brownii* |  | Xing & Cui, 2018 |
| AB763348 | *Ganoderma carnosum* | Nepal: Phulchoki Mountain | Doi et al., Direct Submission |
| EU486458 | *Ganoderma carnosum* | Canada | Denis and Berbee, Unpublished |
| MG654148 | *Ganoderma curtisii* | USA: FL | Loyd et al., 2018 |
| MG654150 | *Ganoderma curtisii* | USA: FL | Loyd et al., 2018 |
| MG654152 | *Ganoderma curtisii* | USA: FL | Loyd et al., 2018 |
| MG654153 | *Ganoderma curtisii* | USA: GA | Loyd et al., 2018 |
| MG654154 | *Ganoderma curtisii* | USA: NC | Loyd et al., 2018 |
| MG654155 | *Ganoderma curtisii* | USA: NC | Loyd et al., 2018 |
| MG654156 | *Ganoderma curtisii* | USA: FL | Loyd et al., 2018 |
| MG654157 | *Ganoderma curtisii* | USA: NC | Loyd et al., 2018 |
| MG654163 | *Ganoderma curtisii* | USA: FL | Loyd et al., 2018 |
| AB811848 | *Ganoderma lingzhi* | Nepal: Lalitpur, Mt. Phulchoki | Hai Bang et al., 2014 |
| AB811852 | *Ganoderma lingzhi* | Nepal: Kathmandu, Dawachok | Doi et al., Unpublished |
| KR093032 | *Ganoderma lingzhi* | Malaysia | Goh et al., Direct Submission |
| LC090753 | *Ganoderma lingzhi* | Japan | Yhiya et al., unpublished |
| MG457485 | *Ganoderma lingzhi* | India | Zothanzama and Zohmangaiha, Unpublished |
| MH160076 | *Ganoderma lingzhi* |  | Loyd et al., 2018 |
| MH160079 | *Ganoderma lingzhi* |  | Loyd et al., 2018 |
| MH160080 | *Ganoderma lingzhi* |  | Loyd et. al 2018 |
| MH160082 | *Ganoderma lingzhi* |  | Loyd et al., 2018 |
| MH160085 | *Ganoderma lingzhi* |  | Loyd et al., 2018 |
| FJ940919 | *Ganoderma lucidum* | China | Xie et al., 2010 |
| GU213471 | *Ganoderma lucidum* | China | Huang et al., unpublished |
| GU213476 | *Ganoderma lucidum* | China | Huang et al., Direct Submission |
| GU213478 | *Ganoderma lucidum* | China | Huang et al., unpublished |
| GU213484 | *Ganoderma lucidum* | China | Huang et al., unpublished |
| GU213485 | *Ganoderma lucidum* | China | Huang et al., unpublished |
| GU213487 | *Ganoderma lucidum* | China | Huang et al., unpublished |
| KX589244 | *Ganoderma lucidum* | China | Zhang et al., 2017 |
| MF476198 | *Ganoderma lucidum* | China | Qaing., Direct Submission |
| MF476199 | *Ganoderma lucidum* | China | Qaing., Direct Submission |
| MG654066 | *Ganoderma lucidum* | USA: CA | Loyd et al., 2018 |
| MH160071 | *Ganoderma lucidum* |  | Loyd et al., 2018 |
| MG654067 | *Ganoderma lucidum* |  | Loyd et al., 2018 |
| MG654070 | *Ganoderma lucidum* |  | Loyd et al., 2018 |
| MG654071 | *Ganoderma lucidum* |  | Loyd et al., 2018 |
| MG654072 | *Ganoderma lucidum* |  | Loyd et al., 2018 |
| MG654073 | *Ganoderma lucidum* |  | Loyd et al., 2018 |
| MG654187 | *Ganoderma meredithae* | USA: FL | Loyd et al., 2018 |
| KY643750 | *Ganoderma mizoramense* | India: Mizoram | Held et al., Direct Submission |
| JQ781874 | *Ganoderma multipileum* | China | Cao et al., 2012 |
| LC149613 | *Ganoderma multipileum* | Nepal | Tamrakar et al., 2016 |
| MH277958 | *Ganoderma oregonense* | USA: Oregon,Klamath Co. | Frank, Unpublished |
| MG654206 | *Ganoderma ravenelii* | USA: FL | Loyd et al., 2018 |
| MG654207 | *Ganoderma ravenelii* | USA: FL | Loyd et al., 2018 |
| MG654208 | *Ganoderma ravenelii* | USA: FL | Loyd et al., 2018 |
| MG654219 | *Ganoderma sessile* | USA: NJ | Loyd et al., 2018 |
| MG654311 | *Ganoderma sessile* | USA: LA | Loyd et al., 2018 |
| MG654312 | *Ganoderma sessile* | USA: MO | Loyd et al., 2018 |
| JQ781878 | *Ganoderma sichuanense* | China | Cao et al., 2012 |
| KT693254 | *Ganoderma sichuanense* | USA | Raja et al., 2017 |
| KP012934 | *Ganoderma sp. MEL 2382607* | Australia: NT | Bonito et al., unpublished |
| KP012964 | *Ganoderma steyaertanum* | Australia: NT | Bonito et al., unpublished |
| JQ781880 | *Ganoderma tropicum* | China | Cao et al., 2012 |
| KJ146707 | *Ganoderma tsugae* | Canada: British Columbia | Berbee et al., Direct Submission |
| MH277960 | *Ganoderma tsugae* | USA: New York,Jefferson Co. | Frank, Unpublished |
| MH277961 | *Ganoderma tsugae* | USA: Vermont,Chittenden Co. | Frank, Unpublished |
| KY646216 | *Ganoderma tuberculosum* |  | Loyd et al., Direct Submission |
| MG654366 | *Ganoderma tuberculosum* | USA: FL | Loyd et al., 2018 |
| KT952361 | *Ganoderma wiiroense* | Ghana: Wiiro | Otto et al., Unpublished |
| KP012910 | *Perenniporia sp. 1 GMB-2014* | Australia: NT | Bonito et al., unpublished |
| MG722742 | *Pilatoporus ibericus* | Georgia | Psurtseva et al., Unpublished |
| JQ312165 | *Polyporales sp. 3 SR-2012* |  | Lakhal et al., Unpublished |
| JQ312182 | *Polyporales sp. 4 SR-2012* |  | Lakhal et al., Unpublished |
| JQ312162 | *Polyporales sp. 4 SR-2012* |  | Lakhal et al., Unpublished |
| JQ312166 | *Polyporales sp. 4 SR-2012* |  | Lakhal et al., Unpublished |
| JQ312204 | *Polyporales sp. 4 SR-2012* |  | Lakhal et al., Unpublished |
| FR750674 | *uncultured Ganoderma* | Germany:Thuringia | Krüger et al., 2012 |

**^1^**listed in Genbank

**^2^**if no location was listed in Genbank location is left blank
