# Supplementary material for: The ITS region provides a reliable DNA barcode for identifying reishi/lingzhi (Ganoderma) from herbal supplements
Source: PLoS One. 2020 Nov 12;15(11):e0236774. doi: 10.1371/journal.pone.0236774 (PMC7660467; doi:10.1371/journal.pone.0236774)
Supplement: S3 Table — Identical sequences from the same species were removed to compress the alignment and facilitate phylogenetic analysis. (DOCX) [file pone.0236774.s004.docx]

**S3 Table. Identical Genbank accessions collapsed to a single representative for phylogenetic analysis.**

| Tree/Alignment Representative | Identical sequences |
| --- | --- |
| MF476200 | EU498091, FJ379262, FJ940919, JN008869, JN008870, JQ781856, JQ781861, MF416197, MF478198, MF476201, KY364244, KY364248, GU213483 2 |
| MH160071 | MG654067, MG654070, MG654071, MG654072, MG654073 |
| JQ781870 | JQ781869, JQ520169, KY364250 |
| JQ781868 | JQ781867 |
| FJ501553 | KX589246 |
| KJ143914 | JQ781874 |
| GU213477 | GU213476, GU213476 |
| JQ781862 | JQ781861, KX055563, KX055562, KX055548 |
| JQ781859 | JQ781858, KX05547 |
| JQ781852 | KJ143912 2 |
| JQ781870 | JQ781869, KY364250 |
| KU310901 | KR093032, KR093032 |
| FJ379262 | KX589250, GU213483, GU213481, GU213479 |
| JN222425 | KX589245, MF476198, EU021456 |
| KM269294 | GU213484 |
| JQ781855 | KY364247 |
| GU213485 | KF146177 |
| GU213480 | HQ235630 |
| HQ235632 | KX05544 |
| KY244063 | KY244068 |
| KY708881 | KY708881 2 |
| KY244065 | KT693253 |
