# Supplementary material for: The ITS region provides a reliable DNA barcode for identifying reishi/lingzhi (Ganoderma) from herbal supplements
Source: PLoS One. 2020 Nov 12;15(11):e0236774. doi: 10.1371/journal.pone.0236774 (PMC7660467; doi:10.1371/journal.pone.0236774)
Supplement: S4 Table — (DOCX) [file pone.0236774.s005.docx]

**S4 Table. DNA concentration and purity for herbal supplement powder samples and fresh samples.**

| **Sample** | **DNA Concentration (ng/uL)** | **Purity (260/280nm)** |
| --- | --- | --- |
| Powder #1 | 5.3 | 0.97 |
| Powder #2 | 3.9 | 0.93 |
| Powder #3 | 12.7 | 1.375 |
| Powder #4 | 15.25 | 1.205 |
| Powder #5 | 28.35 | 1.65 |
| Powder #6 | 175.15 | 0.655 |
| Fresh #1 | 25.1 | 1.91 |
| Fresh #2 | 34.4 | 1.77 |
| Fresh #3 | 7.0 | 1.61 |
