# Supplementary material for: The ITS region provides a reliable DNA barcode for identifying reishi/lingzhi (Ganoderma) from herbal supplements
Source: PLoS One. 2020 Nov 12;15(11):e0236774. doi: 10.1371/journal.pone.0236774 (PMC7660467; doi:10.1371/journal.pone.0236774)
Supplement: Attachment — Submitted filename: Review.docx [file pone.0236774.s006.docx]

The manuscript PONE-D-20-21583 ‘The ITS region provides a reliable DNA barcode for identifying reishi/lingzhi (Ganoderma) from herbal supplements’ presents the results of sequencing ITS rDNA from commercial samples of powdered *Ganoderma* and several fresh control specimens, and compares two different downstream analyses to identify the sequences obtained: distance-based BLAST searches in public databases, and likelihood-based phylogenetic analyses. Unfortunately, I think that the manuscript does not provide enough scientific or technical novelties to be published in PLOS One. ITS rDNA is already known to discriminate most species of *Ganoderma*, and it has been already tested to identify powdered commercial samples. The most relevant result obtained is that the commercial samples analyzed are all mislabeled, suggesting that many marketed products need to be checked. This is important from a commercial point of view, but does not constitute a relevant scientific or technical novelty.

Therefore, I recommend the editors to reject the manuscript. I suggest the authors to look for a different journal to publish this work, or else explore other issues that could provide more interesting results in order to submit it again to PLOS One:

1) It would be great if the authors could quantify the different DNAs present in the samples by means of NGS, in order to estimate the amount of target fungus, additives and contaminants.
2) Another interesting issue is the great amount of misidentifications in public databases (what about UNITE?), and the need for reference sequences (preferably obtained from type collections) to stabilize the taxonomy of these species and provide more accurate database-guided identifications.
3) Authors could focus also on the comparison between distance-based and likelihood-based phylogenetic methods, and discuss cases where both methods produce different results (maybe comparing Ganoderma with other marketed species, i.e. Morchella?).

Below, I provide some more comments about the text:

Keywords = I would shorten the list of keywords. Please remove the words already included in the title. Please replace ‘nuclear ribosomal internal transcribed spacer region’ for ‘ITS nrDNA’. Correct ‘reishi’ (or remove it, as it is present in the title)

Abstract = The first 10 lines could be moved to the Introduction, or substantially reduced to one or two sentences. Please use cursive for species names.

‘clearly identified the predominant fungal DNA was G. lingzhi’ = the PCR product is supposed to belong to the predominant DNA present in the sample, but this could not be the case, as genus-specific primers were employed. Other species could be present and not amplified because of the primer specificity. You can change the sentence to ‘clearly identified the presence of G. lingzhi DNA’. Did you obtain a mixed signal after sequencing the PCRs done with universal primers?

‘ITS is a successful and cost-effective method for DNA-based species authentication’ = ITS is not a method, but a region of the rDNA. ‘ITS-based phylogenetic analysis is a successful and cost-effective method for DNA-based species authentication’ would be more correct.

Introduction

‘Barcoding is an efficient molecular tool’ --> better ‘Molecular barcoding is an efficient tool’

‘identifying morphologically, anatomically and biochemically enigmatic samples’ = Anatomy is a specific type of morphological study. Maybe you mean ‘macroscopically, microscopically and biochemically enigmatic samples’? Please correct other similar sentences.

‘especially helpful for maintaining the validity of active ingredients’ = DNA barcoding can detect the presence/absence of some target taxa, but it does not provide any info about the activity of ingredients. DNA of a species can be present after losing the biological activity of its compounds (i.e. after an aggresive processing).

‘avoiding contaminants that may cause allergic reactions for consumers’ = other species can be present also as additives. DNA testing can be employed also to check for their presence.

‘Reishi’ = please add a latin binomial with authors when this species is mentioned for the first time, or at least *Ganoderma* spp. if the vulgar name is applied to more than one species. Please add the authors of all scientific names when they are first mentioned.

‘The G. lucidum clade consists of several species that are in taxonomic flux’ = maybe better ‘The G. lucidum clade includes several phylogenetic lineages that received an unstable taxonomic treatment in the past’.

‘Ganoderma lucidum sensu lato (including G. ‘tsugae’)’ = Ganoderma tsugae is a valid name, so it does not need quotation marks. You can correct it as ‘(including G. tsugae Murrill)’.

‘can be found in the wild from Europe to northeastern China (likely escaped from cultivation in California and Utah, see [22])’ = there are three lineages within the G. lucidum clade (G. lucidum s. str., G. tsugae and G. oregonensis). Do you mean that G. tsugae and G. oregonensis could have escaped from cultivation? Or maybe you refer to Ganoderma luciudm s. str. instead?

‘According to several recent molecular phylogenetic studies, the taxonomy of G. lucidum and G. lingzhi remains uncertain [18, 26, 31].’ = I disagree, both species can be easily discriminated genetically. The whole paragraph needs to be corrected.

‘The nuclear ribosomal ITS region is a powerful tool for barcoding’ = better ‘the nuclear ribosomal internal transcribed spacer region (ITS) is an informative DNA region for barcoding...’

‘lineage-specific primers have been developed for many groups of fungi in order to improve PCR success’ = in many cases (especially for rDNA) the lineage-specific primers were not designed to improve PCR success, but to provide diagnostic primers to check the presence/absence of some species, or else to avoid the amplification of contaminant organisms.

‘Lineage-specific primers improve PCR success especially when working with compromised DNA templates that may be degraded, contain inhibitors, or be composed of a mixture of species’ = If DNA is degraded, specific primers by themselves will not improve PCR success. Maybe this could happen as a consequence of a different PCR approach, a smaller amplicon, or other factors unrelated with primer specificity. However, lineage-specific primers can improve PCR success when designed for non-conserved annealing regions if the ‘universal’ primers available do not work. In case that inhibitors are present, specific primers will not provide any improvement to PCR success. If a mixture of species is present, then specific primers can bypass the contaminants, improving PCR specificity (but not PCR success, which was also successful with the universal primers).

‘The two main approaches for analysis of the DNA sequences arising from barcoding investigations are similarity-based measures (e.g., best-hit BLAST or nearest neighbor analysis) and phylogenetic methods (e.g., maximum likelihood or Bayesian tree-building algorithms).’ = ‘distance’ is usually employed instead of ‘similarity’. You probably refer to genetic distance, but other types of distances can be measured, so please specify ‘genetic distance’. In addition, distance-based methods are employed also to build phylogenies, so they are phylogenetic methods. You could maybe call the second kind ‘likelihood-based methods’, since ML and bayesian approaches make use of the likelihood function.

‘Herein, we present an efficient barcoding method for unambiguous identification of the herbal supplement, reishi (G. lingzhi).’ = barcoding refers to the sequencing itself, but the identification needs also some kind of analysis, so maybe better ‘‘Herein, we present an efficient method for unambiguous identification of the herbal supplement, reishi (G. lingzhi).’

‘All powdered samples are morphologically unidentifiable as a mushroom’ = did you check for the presence of spores?

Table 1

I think it would be better to hide the name of stores and suppliers, unless you have the explicit consent of these companies to publish the results of your study. The species name is the one provided by the seller, or the results obtained from your analyses? Please clarify.

DNA extraction

‘Each nutritional supplement was extracted twice’ = you mean that two subsamples were taken from each sample and DNA was extracted from each of them, right? Please clarify.

‘Ganoderma-specific primers (G-ITSF1 and G-ITS-R2) were selected based on consistently producing strong single bands’ = you mean that the other primers produced multiple bands or weak bands?

‘These primers were designed to prevent amplification from plant or other fungal DNA, which is a common problem with herbal supplements since they often include a plant-based growing medium.’ = what about the fungal-specific primer ITS1F and the basidiomycete-specific primers ITS4B? Is contamination with other fungi a real issue in commercial samples of powdered Ganoderma?

‘ethidium bromide’ = I strongly recommend you to replace ethidium bromide with GelRed or other less toxic and contaminating DNA stain.

‘Forward and reverse chromatograms for each sample were trimmed to remove primer sequence and low quality sequence’ = chromatograms are trimmed to remove low quality reads. Primer sequences are rarely reached by the chromatogram, and they are almost always poorly resolved. However, they can be recovered in some cases. Also, you should correct ambiguous reads due to noise, dye blobs, and heteromorphic sites. So the most correct would be to say ‘Forward and reverse chromatograms for each sample were trimmed to remove low quality reads at the extremes, and edited to correct ambiguous reads and heteromorphic sites between them.’

BLAST = from which platform did you launch BLAST algorithm? Please cite Cochrane et al. (2011) if accessed from INSDC.

‘We also added all the unique Reishi samples (G. lingzhi and G. lucidum) of ITS using ENTREZ’ = what do you mean by ‘all the unique Reishi samples’? I dont understand, please clarify.

‘Finally, we included representatives of as many Ganoderma species we could find using a filtered discontiguous megablast’ = Maybe this is not the case, but there could be sequences related to your samples that are not listed in the BLAST results, especially if some species are overrepresented in GenBank. Maybe you could have just included those species more closely related to your samples by checking the phylogenetic studies available.

‘Several outgroup sequences were chosen’ = the outgroup should be selected from the clade most closely related to the sequences to be analyzed. In your case, this could be another species of Ganoderma, or the type of a sister genus.

‘Bayesian analysis used 1,500,000 Markov chains’ = usually 4-6 chains are employed. You probably mean 1.5 M generations.

‘and after a burn-in length of 750,000 samples.’ = 1.5 M generations sampled each 750 generations make a total 2000 sampled trees. So, you cannot remove 750.000 samples. You probably mean that you removed the samples taken during the first 750.000 generations (1000 samples, a 50% burn-in).

‘All primer pairs produced visible bands of expected size for the ITS region for both fresh and powdered samples. The Ganoderma-specific primers were chosen for all other analyses.’ = you should explain why these primers are chosen instead of the universal ones. Did you find problems in the sequences produced by the universal primers? Were they chosen to avoid putative contaminants? Please remove cursive from ITS.

‘Nucleotide sequences were recovered from the ITS region from all of the samples.’ = this sentence is superfluous. You should remove it and reorganize the paragraph.

‘After trimming the sequences, lengths ranged from 780 base pairs to 895 base pairs with an average of 854 base pairs.’ = you probably refer to the sequences obtained from GenBank, but it seems like you speak about the sequences produced de novo. Please clarify.

‘31 branches (36%) were greater than 70%’ = 70% is the bootstrap support, please specify it.

Fig. 1 = it would be better to show a phylogram instead of a cladogram. Also, you could add bayesian PP support to the nodes.

‘Fresh #3 had a sister relationship (to “G. lucidum” KX589244)’ = it is better so say ‘a significant relationship’. Change also in the following sentence. These relationships are based on very few bases from a single marker, so they should be interpreted cautiously.

‘Ganoderma brownii and falls clearly outside the G. lingzhi clade in a poorly resolved cluster of Ganoderma accessions in Clade A’ = why none of the two G. brownii ITS sequences in GenBank appear in the tree? Probably due to the sampling procedure. You could have ordered BLAST results by % similarity (removing those <50% coverage).

‘phylogenetically aberrant Genbank accessions’ = maybe better ‘putatively incorrectly named GenBank accessions’

‘a G. lucidum sample (MG654066) falls within a small, yet moderately supported clade of mostly North American samples’ = but in Fig. 1 you report that ‘The red rectangle [MG654066] identifies the only true G. lucidum sample per Loyd et al. [14]’ So, this is not ‘a phylogenetically aberrant Genbank accession’

‘Our study demonstrates that the ITS region provides an efficient barcode for store-bought

reishi herbal supplements as previously described by Loyd et al. [14] and Raja et al. [38].’ = maybe better to say that your study supports the conclusions reached by earlier authors.

‘Clade A sensu Zhou et al. [24] and Loyd et al. [14] which only includes “G. lucidum” as defined in the broadest sense’ = this is very confusing. Clade A sensu Loyd et al. includes multiple species, but not G. lucidum. You could say that clade A includes species morphologically similar to G. lucidum. A sensu lato always includes the sensu stricto plus other clades.

‘Technically all of our samples are misidentified since they are being sold as “G. lucidum”, yet are molecularly allied with the G. lingzhi samples in Clade A.’ = So, MG654066 is not an aberrant accesion, but the correct concept of G. lucidum.

‘Genbank sample MG654066 named G. lucidum (in Clade B) is the only sample that represents G. lucidum sensu stricto’ = this seems to mean that MG654066 is the only known sequence of G. lucidum s. str. However, there are others (at least 8 more in Loyd et al.). It is the only one in your tree, probably because of the sampling process employed.

‘there were distinct phylogenetic affinities clearly indicating separate sources’ = this could be true, but not necessarily.

‘Our results were generally robust to whether we used the entire ITS region or the trimmed region’ = not for G. brownii. This should be enough to say that the sampling method employed to obtain closely related sequences from GenBank was not the most suitable one. I think you should have ordered GenBank results by their similarity with the query, not the BLAST score.
